# Interplay between ubiquitination and ADP-ribosylation and the case of dual modification ADPr-Ub

**DOI:** 10.1042/EBC20253040

**Published:** 2025-10-09

**Authors:** Kang Zhu, Chatrin Chatrin, Rebecca Smith, Dragana Ahel, Ivan Ahel

**Affiliations:** 1 Health Science Center, East China Normal University, Shanghai, 200241, China; 2 Sir William Dunn of Pathology, University of Oxford, South Parks Road, Oxford, OX1 3RE, U.K

**Keywords:** ADP-ribosylation, DNA damage, immunity, PARP, ubiquitin

## Abstract

Ubiquitination is a fundamental post-translational modification essential for nearly all cellular activities. Traditionally, ubiquitination has been understood as a protein modification, where ubiquitin (Ub) molecules are covalently attached to the lysine residues of substrate proteins, thereby modulating their function, localization, or degradation. However, recent discoveries have expanded the scope of ubiquitination beyond protein substrates. One of the examples is ubiquitination of ADP-ribose moieties on proteins or nucleic acids that leads to the formation of a dual-hybrid modification ADP-ribose-Ub (ADPr-Ub). This novel form of ubiquitination is catalyzed by Deltex ubiquitin ligases that act in concert with PARPs (Poly (ADP-ribose) polymerases), enzymes modifying their substrates by ADPr modification. This review summarizes our current knowledge of mechanisms and potential functional implications of ADPr-Ub. We also cover other examples of the interplay between ADP-ribosylation (ADPr) and ubiquitination beyond Deltex enzymes and ADPr-Ub.

## Introduction

**Figure 1:**
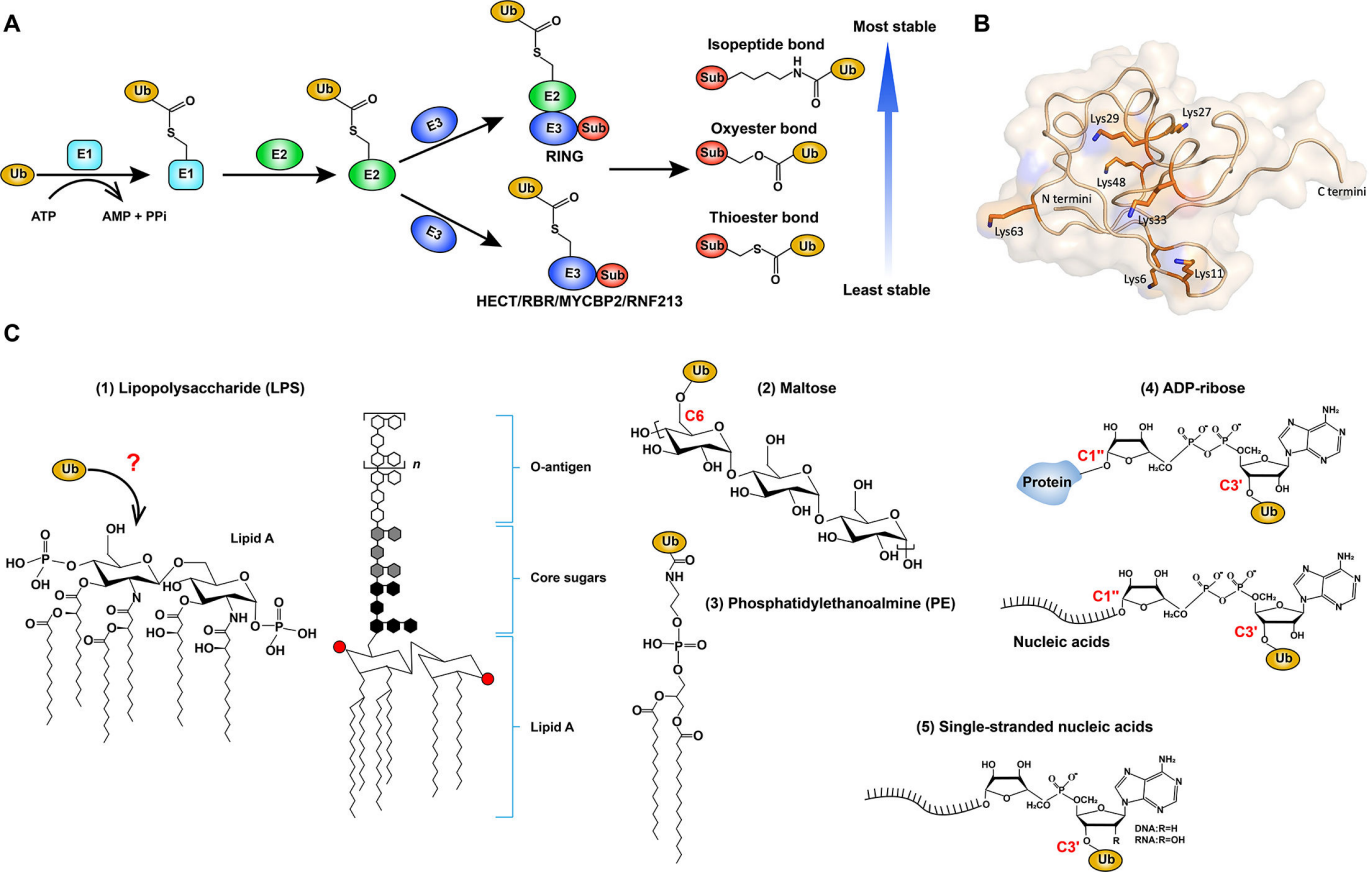
Canonical ubiquitination and non-protein ubiquitination. (**A**) Schematic image of the ubiquitination cascade. Ubiquitination is a stepwise enzymatic process. Ubiquitin (Ub) is first activated by the E1 enzyme in an ATP-dependent manner and then transferred to an E2 conjugating enzyme. Finally, Ub is attached to a substrate protein either directly via a RING-type E3 ligase or through a HECT/RBR-type/transthiolating (MYCBP2/RNF213) E3 ligase that forms an intermediate Ub-E3 conjugate before substrate transfer. (**B**) Structure of Ub (PDB: 1UBQ) showing the positions of all seven lysine residues (colored in orange). (**C**) Non-protein substrates of ubiquitination: [1] The lipid A moiety of LPS on the surface of Salmonella [2]; Glucosaccharides such as maltose [3]; Phosphatidylethanolamine [4]; ADP-ribose, either free or conjugated to protein/nucleic acids [5]; Single-stranded nucleic acids. E1, ubiquitin-activating enzyme; E2, ubiquitin-conjugating enzyme; E3, ubiquitin ligase; LPS, lipopolysaccharide; Sub, substrate; Ub, ubiquitin.

Ubiquitination is a complex and highly regulated post-translational modification (PTM) that plays a critical role in regulating protein stability, cellular signaling, and various physiological processes [1]. This multi-step enzymatic process involves a cascade of three enzymes: E1 (ubiquitin-activating enzymes), E2 (ubiquitin-conjugating enzymes), and E3 (ubiquitin ligases) [2]. E1 enzymes initiate the process by activating ubiquitin (Ub) in an ATP-dependent manner, forming a high-energy thioester bond between the Ub C-terminal glycine and the active site cysteine of E1 [3–5]. The activated Ub is then transferred to an E2 enzyme, which subsequently collaborates with an E3 ligase to facilitate substrate specificity and catalyze the covalent attachment of Ub to a target protein [6] (Figure 1A). Moreover, with seven lysine residues (K6, K11, K27, K29, K33, K48, and K63) and the N-terminal methionine residue (M1) available as anchoring sites, Ub can form various chain types or mixed Ub chains with diverse topologies, enabling versatile regulatory functions in cellular processes, which is referred to as the ‘ubiquitin code’ [7]. The fate of the ubiquitinated protein depends on the type of Ub linkage formed. K48-linked polyubiquitin chains serve as a classical signal for proteasomal degradation, ensuring the controlled removal of misfolded, damaged, or unneeded proteins [8,9]. In contrast, K63- and M1-linked Ub chains primarily mediate non-degradative functions, such as immune signaling transduction [10,11], autophagy [12], and endocytic trafficking [13]. Other types of Ub linkages, including K6, K11, K27, K29, and K33 (Figure 1B), are less well understood but are increasingly recognized for their roles in cellular signaling and regulation [14].

E3 ligases are central to the ubiquitination process, as they determine substrate specificity and catalyze the final step of Ub transfer (Figure 1A). These enzymes are classified into three main families based on their catalytic mechanisms: RING (Really Interesting New Gene), HECT (Homologous to the E6-AP Carboxyl Terminus), and RBR (RING-between-RING) E3 ligases [15–17]. RING-type ligases function primarily as scaffolds, facilitating the direct transfer of Ub from the E2 enzyme to the substrate without forming an intermediate. HECT-type ligases, in contrast, form a transient ubiquitin-thioester intermediate on a catalytic cysteine residue before transferring Ub to the substrate. RBR ligases combine features of both RING and HECT families, employing a two-step mechanism in which the first RING domain recruits an E2 enzyme, and the second RING domain contains a catalytic cysteine for Ub transfer [17]. Recently, two additional new classes of transthiolating E3 have been discovered. MYCBP2 (Myc-binding protein 2), representing the RCR (RING-Cys-Relay) class, utilizes two catalytic cysteine residues to facilitate the internal Ub transfer [18]. Mysterin/RNF213 utilizes a catalytic cysteine in a non-canonical RZ-finger (RNF213–ZNFX1 finger) domain to conjugate Ub to substrates [19,20]. Given their regulatory control over ubiquitination, E3 ligases are frequently implicated in human diseases, including cancer, neurodegeneration, and immune disorders, making them attractive therapeutic targets [21].

Historically, ubiquitination research has focused primarily on protein substrates, given its well-established role in protein quality control and degradation. However, recent discoveries have expanded this paradigm by demonstrating that Ub can also be covalently attached to non-protein biomolecules [22–25]. This emerging area of research, termed non-protein ubiquitination, has revealed novel physiological functions that extend beyond protein turnover. For instance, lipopolysaccharide (LPS) on the surface of *Salmonella* was identified as the first non-protein ubiquitination substrate, which is modified with Ub chains by host E3 RNF213 during infection [22]. This modification recruits the linear ubiquitin chain assembly complex (LUBAC), activating cell-autonomous immune signaling pathways to accelerate pathogen clearance. Similarly, ubiquitination of phosphatidylethanolamine (PE) on endo/lysosomal membranes in cells has been shown to be significantly elevated under stress-inducing conditions, including nitrogen starvation, mTOR inhibition, or lysosomal damage, indicating its functional role in mediating cellular adaptation to metabolic and organelle stress [25]. Another notable example involves HOIL-1 (haem‐oxidised IRP2 ubiquitin ligase‐1), a LUBAC component, which ubiquitinates glycogen. Specifically, mice harboring HOIL-1 ligase-inactivating mutations accumulated toxic polyglucosan aggregates in brain and cardiac tissues, suggesting that HOIL-1 mediates an intracellular glycan quality control mechanism to prevent harmful polysaccharide deposition [24]. Recently, ubiquitination of nucleic acids has been suggested [26,27] (Figure 1C). Finally, Deltex family E3 ligases were found to ubiquitinate pre-existing ADP-ribose modifications on proteins and nucleic acids (originally formed by PARPs), creating dual ADPr-Ub modification (also referred to as MARUbylation) [23,28,29], which will be discussed in detail below (Figure 1). This review will cover the interplay between ubiquitination and ADP-ribosylation, with a particular focus on ADPr-Ub, first describing its discovery and summarizing the biochemical properties and molecular mechanism of ADPr ubiquitination, then predicting the potential roles of its resultant hybrid ADPr-Ub modification in cancer, immune signaling, and protein homeostasis, while highlighting its significance in cellular function and molecular signaling networks.

## Interplay of ubiquitination and ADPr

Ubiquitination is known to have interplay with other PTMs, including ADPr [30–32]. ADPr modification is found in all kingdoms of life [33]. It involves the transfer of ADP-ribose moieties from nicotinamide adenine dinucleotide (NAD^+^) to target proteins or nucleic acids [34–40] and regulates many processes such as DNA damage repair, immune response, and bacterial metabolism [34,41,42]. The largest family of enzymes synthesizing ADPr in humans are PARPs, which list 17 family members [43,44]. Like for the ubiquitination system, ADPr can come as mono-, poly-chains, or even branches of repeating ADPr units [34]. Most PARPs only catalyze mono-ADPr of substrates [45]; however, PARP1/PARP2 (involved in DNA damage repair) and tankyrases 1/2 (PARP5a and PARP5b) can form long poly-ADPr (PAR) chains that can occasionally branch via O-glycosidic at the C2 position of ribose moieties [34,46,47]. ADPr is a reversible and dynamically regulated PTM, tightly controlled by specialized hydrolases [48]. PARG (Poly(ADP-ribose) glycohydrolase) can hydrolyze PAR and branched chains [49–52], while there are numerous mono-ADPr-hydrolases specific for different amino acid linkages. For example, (ADP-ribosylhydrolase 3) ARH3 primarily acts on serine-linked ADPr formed in DNA damage response (DDR) [53], while TARG1 acts on glutamates/aspartate-linked ADPr in proteins and nucleic acids [54–57]. Proteins modified by ADPr undergo functional regulation by alteration of their activity/stability or localization. The ADPr modification is read through non-covalent interactions by other effector proteins that possess specialized ADPr-recognition modules [58]. Among these, the PAR-binding zinc fingers (PBZ) domain [59,60], macrodomains [61–63], and WWE domain (named after the three conserved residues tryptophan (W), tryptophan (W), and glutamate (E)) [32] are well-characterized examples whose interaction has been resolved through structural studies.

**Figure 2:**
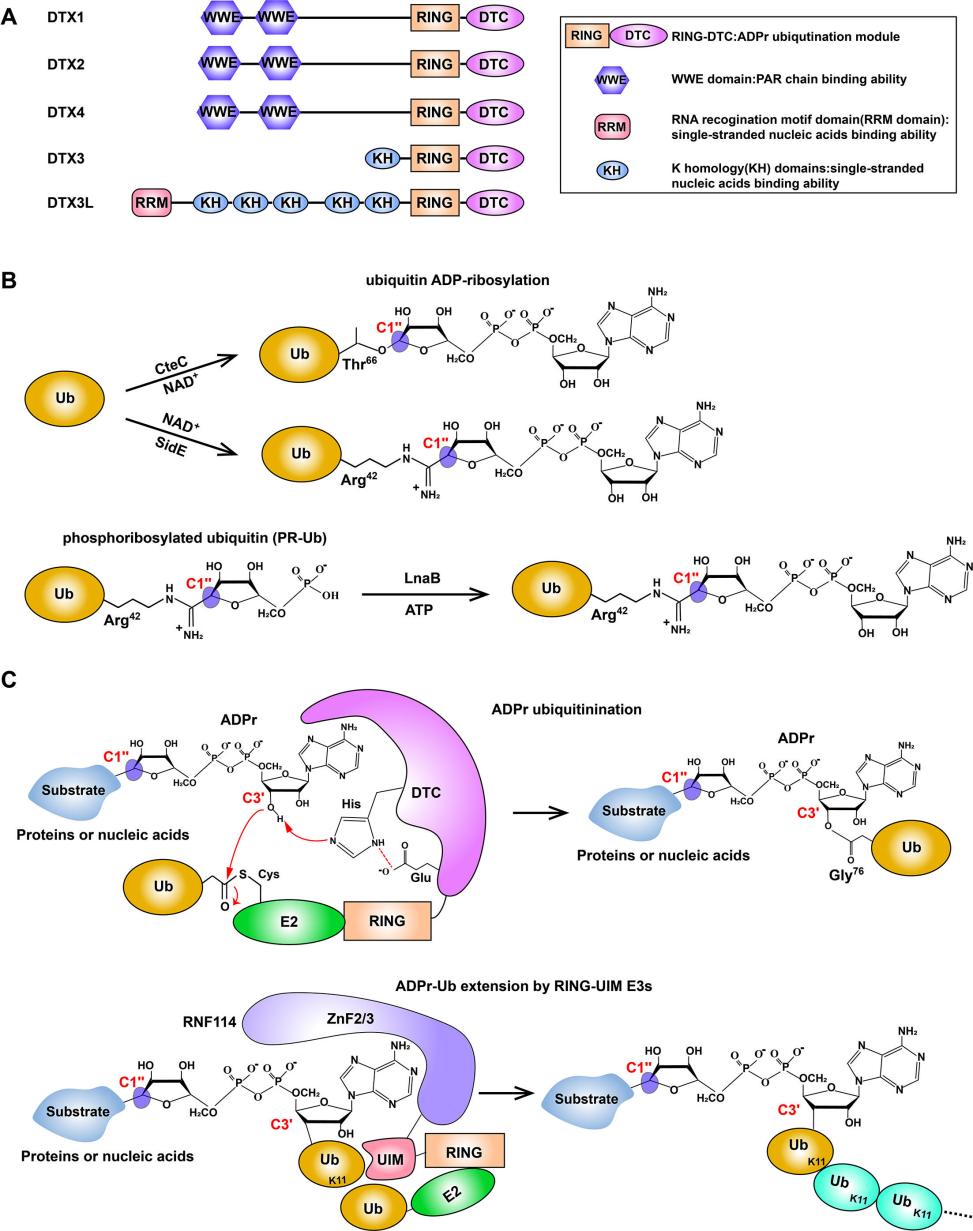
Molecular mechanism of ADPr ubiquitination by Deltex family E3s. (**A**) Domain architecture of Deltex family E3 ligases; (**B**) Schematic illustration of Ub ADP-ribosylation catalyzed by the bacterial effector proteins SidE (*Legionella*) and CteC (*Chromobacterium*) and LnaB (*Legionella*)-mediated formation of Ub-ADPr from AMPylation of phosphoribosylated ubiquitin (PR-Ub). (**C**) Mechanistic model of ADPr ubiquitination catalyzed by the shared RING-DTC fragment of Deltex family E3 ligases. The resulting ADPr-Ub hybrid modification can subsequently be extended by RING-UIM family E3 ligases (e.g. RNF114 and RNF166), which utilize their ZnF and UIM domains to specifically recognize ADPr-Ub and catalyze the elongation of K11-linked ubiquitin chains. UIM, ubiquitin interacting motif; ZnFs, zinc fingers; RRM, RNA recogination motif domain; KH, K homology domain.

The WWE domain is particularly interconnected with ubiquitination systems [64]. Notably, the WWE domain is conserved in multiple human Ub E3 ligases, including RNF146, Deltex family members (DTX1 (Deltex 1) , DTX2 (Deltex 2) , and DTX4 (Deltex 4), HUWE1 (HECT, UBA, and WWE domain containing protein 1) , and TRIP12 (thyroid hormone receptor interactor 12) [65–68]. Multiple macrodomains are found in PARP9 and PARP14 that are part of the heterodimer complex with the DTX3L [69]. CHFR (Checkpoint protein with FHA and RING finger domains), which is a Ub E3 ligase involved in mitotic checkpoint control, possesses a PBZ module [59,66,70]. Conversely, PARPs themselves sometimes encode Ub-related domains, as highlighted by PARP10’s ubiquitin-binding motifs [71] and PARP6/8’s enigmatic E2-like folds [44], whose functional roles remain to be described. The best understood example of the interplay between ubiquitination and ADPr signaling is the PAR-dependent ubiquitylation mechanism mediated by tankyrase 1/2 (PARP family members) and RNF146. Tankyrase 1/2-mediated PARylation has been shown to prime RNF146-dependent ubiquitination of substrate proteins, marking them for proteasomal degradation – a paradigm of PAR-directed protein turnover (e.g. Axin degradation in Wnt signaling) [72–74]. In bacterial pathogens, ADPr enzymes are used to manipulate host ubiquitination pathways. For instance, the *Legionella pneumophila* effector SidE (substrate of Icm/Dot effector) family members (SidE and its homologs SdeA, SdeB, and SdeC (SidE derivatives A–C)) hijack ubiquitination through a non-canonical mechanism [75,76]. Specifically, SidE enzymes catalyze ADPr of Ub at Arg42 via their mono-ADP-ribosyltransferase domain (Figure 2B). This is followed by phosphodiesterase-mediated cleavage of AMP to generate a phosphoribose-linked Ub (PR-Ub), which can be subsequently converted back to the ADP-ribosylated Ub by LnaB (*Legionella pneumophila* protein lpg2527) -mediated AMPylation (Figure 2B) [77,78]. This modified Ub is then attached to serine residues of host substrates (e.g. ER structural proteins) via a phosphodiester bond, inducing ER fragmentation and recruitment of membranes to pathogen vacuoles [79]. Similarly, CteC from *Chromobacterium violaceum* mono-ADP-ribosylates Ub at Thr66 (Figure 2B), thereby inhibiting host polyubiquitin chain synthesis, recognition, and removal. This modification ultimately disrupts the host’s antibacterial immune response [80].

Strikingly, the Deltex family E3s have recently expanded the cross-talk between ADPr and Ub after it was discovered that they are capable of synthesizing a dual ADPr-Ub hybrid modification [23,28], which will be elaborated below.

## Discovery of ADPr ubiquitination

The discovery of ADPr-Ub stemmed from the attempts to find the activity for PARP9, a PARP family member with no assigned catalytic activity [44,45]. The PARP9 gene is co-transcribed with the DTX3L E3 ligase gene from a bidirectional promoter and their protein products assemble into a heterodimer [81]. Based on this, the Paschal laboratory investigated the ADPr activity of the PARP9/DTX3L protein complex and detected efficient attachment of a single ADP-ribose unit to the C-terminus of Ub [82]. The authors suggested that PARP9 becomes catalytically competent for Ub ADPr only with the partner DTX3L protein, which would be reminiscent of what was later shown for the PARP1/HPF1 (Histone PARylation Factor 1) complex in modification of proteins involved in DDR [83]. Specifically, just as HPF1 provides a key catalytic residue essential for PARP activity, it was thought that in an analogous way, DTX3L enabled PARP9’s ADPr activity. Interestingly, in the case of PARP9/DTX3L, the ADPr reaction requires not only NAD^+^ but also E1, E2 enzymes, and ATP, which are essential components of ubiquitination. However, a subsequent study by the Huang lab revealed that PARP9 itself does not catalyze Ub ADPr [84]. They showed that the isolated C-terminus of DTX3L, or of other Deltex family members (DTX1, DTX2, DTX3, and DTX4), containing only the DTC (Deltex C-terminus) and RING domains (Figure 2A) can efficiently perform the same reaction conjugating Ub to the ADP-ribose molecule. The study showed that the DTC domain binds NAD^+^ and suggested that the C-terminus of Ub is then ADP-ribosylated at the distal side of ADP-ribose via the conventional C1 position by an unclear mechanism.

Despite these studies, the exact nature of the reaction product and the underlying catalytic mechanism remains unconfirmed and uncharacterized. To investigate the molecular mechanism of Ub ADPr catalyzed by Deltex family members, the Ahel lab reconstituted this reaction *in vitro* using the DTX2-RING-DTC fragment and analyzed the reaction products using high-performance liquid chromatography coupled with mass spectrometry (HPLC-MS) [23]. Surprisingly, the Ub ADPr product reported in previous studies was not detected. Instead, Ub was found to be attached to the proximal (opposite) ribose moiety of ADP-ribose, and NAD^+^ was shown to be the substrate for this reaction^23^. Further investigation using NMR spectroscopy provided direct evidence of ubiquitination occurring on the 3′-hydroxyl group of ADPr [23] (Figure 2C). Altogether, these studies reveal that ubiquitination of ADPr takes place (Figure 2C) rather than ADPr of Ub (Figure 2B). This is highly relevant, as this arrangement allows the formation of a dual, hybrid modification on macromolecules initially modified by ADPr, and the discovery of this novel modification provides new exciting opportunities for such modification to regulate biological processes.

A recent study suggested that PARP9/DTX3L could potentially act to form ADPr-Ub in conjunction with an antiviral PARP, PARP14 [69,85,86]. This hypothesis is supported by the spatial co-occurrence of their respective Ub and ADPr products in cells upon interferon treatment [69]. More recently, the ADPr-Ub dual modification by another PARP family member, PARP10, has been for the first time directly detected in cells [29]. It is not clear which E3 works with PARP10 in this cellular system, but auto-modification of PARP10 and subsequent ADPr-Ub was previously reconstituted using recombinant PARP10 and DTX2 [23]. The ADPr-Ub reaction was also reconstituted on the N-terminal of the histone tail (e.g. Histone 3 [H3]) using DTX2 [23], indicating a potential mechanism by which ADPr-Ub may recruit its readers to DDR sites [87]. Fittingly, protein substrates modified by ADPr-Ub, including histones and PARP1, have been recently identified by mass spectrometry in cells during the DDR [88]. These emerging findings collectively suggest that distinct PARP-E3 ligase pairs may generate ADPr-Ub in a context-dependent manner.

## Mechanism of ADPr ubiquitination

Unlike traditional E3 ligases that target lysine residues on proteins, Deltex E3 ligases can also facilitate the covalent attachment of Ub to ADPr, specifically on the 3′ hydroxyl group of ADPr proximal ribose [23]. While canonical RING-type E3 ligases form complexes with Ub-loaded E2 enzymes but typically do not alter the chemical specificity of the Ub transfer reaction – acting instead to activate the intrinsic reactivity of their partner E2s, Deltex E3 ligases have evolved to have a specialized DTC domain adjacent to the RING domain, which binds NAD^+^ and ADPr [84,89]. Mechanistically, Deltex E3s recruit an E2~Ub conjugate and an ADPr molecule using the RING and DTC domains, respectively [23]. Moreover, the arrangement of the RING and DTC domain is crucial to catalyze the reaction. In all Deltex family E3s, the RING and DTC domains are connected by a short, flexible linker, and altering the linker properties is detrimental to ADPr-Ub formation [84]. Next, the thioester bond between E2 and Ub is juxtaposed to the 3′ hydroxyl group of ADPr proximal ribose due to the flexible linker. The DTC domain contributes one histidine residue and one glutamate residue (His-Glu catalytic triad) to apparently deprotonate and thus encourage the 3′ hydroxyl group to attack the E2~Ub conjugate to accomplish ADPr ubiquitination, promoting nucleophilic attack and subsequent Ub transfer [23]. Interestingly, the proposed arrangement of 3′ OH and His-Glu catalytic triad bears resemblance to the catalytic triad of serine proteases, which consists of linearly arranged hydroxyl, histidyl, and carboxyl moieties, and as a conserved way of activating a serine residue for nucleophilic attack to the isopeptide bond in protein substrates [90]. The molecular mechanism of ADPr-Ub does not involve the conventional formation of a thioester intermediate between the E3 enzyme and Ub, distinguishing it from HECT and RBR E3 ligases [17]. Overall, this represents a novel ubiquitination paradigm, establishing DTX enzymes as the only known RING-type E3 ligases capable of redirecting the chemical specificity of their partner E2s to target hydroxyl groups.

### Ubiquitination of substrates other than mono-ADPr protein substrates

Examples of PARP10 and PARP14 demonstrate that mono-ADPr is enzymatically catalyzed on themselves [45,91,92] and the subsequent ubiquitination of the ADPr moiety by Deltex family E3 generates ADPr-Ub dual modification *in vitro*. In the case of PARP10, ADPr-Ub on itself has been proved to exist in cells [29], and mono-ADP-ribosyl ubiquitination (MARUbylation) as an alternative name has been suggested [23,29,69]. However, ubiquitination of PAR has been demonstrated as well, at least *in vitro* [26,93]. Specifically, DTX1 and DTX2 modify the terminal ADPr unit of the PAR chain. The potential relevance of PAR ubiquitination *in vivo* is further supported by the presence of PAR-binding WWE domains in three Deltex family members (DTX1, DTX2, and DTX4) [26,68 *].*

In addition, there is evidence showing that ADPr-Ub can form on nucleic acids [28]. Some PARP family members, such as PARP1, PARP3, PARP10, PARP11, and PARP14, can target nucleic acids in certain conditions *in vitro* [37,39,40,44,86,94], in particular modifying the phosphate groups at RNA and DNA ends or breaks. Interestingly, both PARP10 and PARP14 have putative RNA binding RRM domains [95] as well as KH domains that have been suggested to mediate both protein–nucleic acids and protein–protein interactions [44,69,86,96,97]. Intriguingly, DTX3L and DTX3 also have KH domain(s) [26,28], and this further supports a possibility that PARP10/PARP14 and DTX3L/DTX3 collaborate to catalyze ADPr-Ub on nucleic acids. However, currently, there is no evidence yet for the existence of ADPr-Ub dual modification on nucleic acids in cells. Taken together, it seems that the Deltex E3s could utilize their PAR-binding domain–WWE domain or nucleic acids binding domain – RRM or KH domains to guide specificity of their ADPr-Ub reactions; however, further investigation is needed to clarify the (patho)physiological roles of these modifications.

## Possible functional implications of ADPr-Ub

Only recently have the first pieces of evidence emerged demonstrating that ADPr-Ub modification exists in cells; however, given the current technical limitations in detecting and characterizing this modification, it remains premature to draw definitive conclusions about its physiological roles. It is anticipated that it would be around processes such as immunity, genome stability, and Notch signaling. Prior studies on Deltex family E3 ligases primarily implicate their roles as negative regulators in the Notch signaling pathway, with functional conservation observed in model organisms such as *Drosophila melanogaste*r [98–100]. While *Drosophila melanogaster* retains a single ancestral Deltex gene, humans exhibit evolutionary expansion of this family through five paralogs: DTX1, DTX2, DTX3, DTX4, and DTX3L. Beyond their conserved roles in Notch signaling, these paralogs exhibit functional divergence: for instance, DTX3L and DTX2 have also been linked to DNA repair [82,101,102].

**Figure 3:**
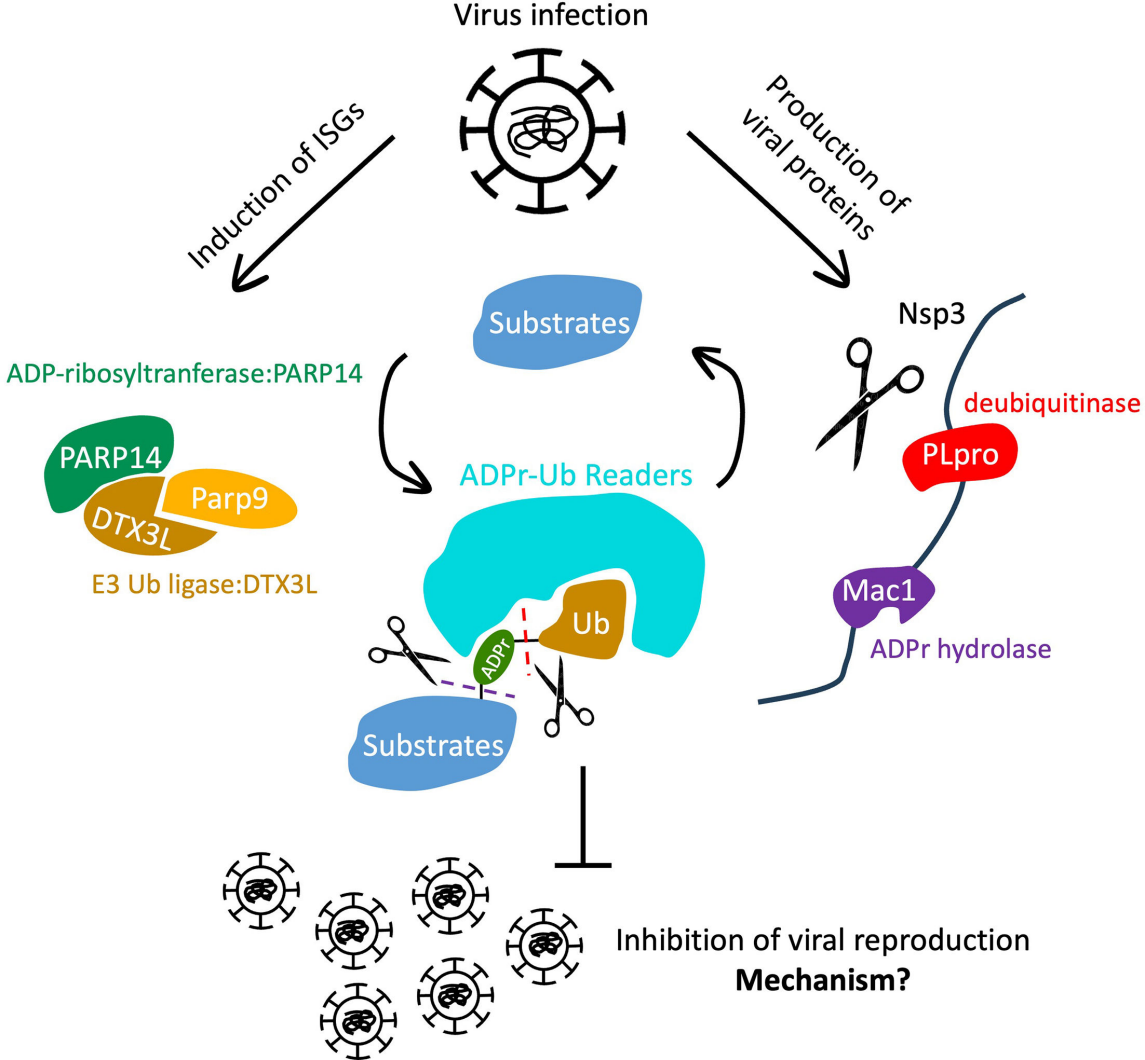
Possible antiviral role of PARP14/DTX3L/PARP9. Interferon (IFN) signaling induced by viral infection up-regulates expression of PARP14, PARP9, and DTX3L, which are encoded from the same genomic locus and likely function together as a complex. This complex harbor both ADP-ribosyltransferase (PARP14) and E3 ubiquitin ligase (DTX3L) activities and may mediate hybrid ADPr-Ub modifications on host or viral proteins or nucleic acids. These modifications could potentially contribute to antiviral defense by altering the stability, activity, or recognition of viral components. SARS-CoV-2 encodes an ADP-ribosylhydrolase (Mac1) and deubiquitinase (PLpro), which may sequentially cleave the ADPr-Ub modification, thus antagonizing host-mediated ADPr-Ub signaling as a strategy to evade immune responses. ISGs, Interferon-Stimulated Gene; Nsp3, Non-structural protein 3; SARS-CoV-2, Severe acute respiratory syndrome coronavirus 2; Mac1, macrodomain; PLpro, Papain-like protease.

In humans, PARP14/DTX3L/PARP9, whose expression is interferon-induced, are expected to be involved in antiviral function [69,85,103]. Importantly, they are all expressed from the same genomic locus, show similar transcriptional profiles, and have fast evolution [104]. This complex contains both an ADP-ribosyltransferase, PARP14, and a E3, DTX3L, and their respective ADPr and ubiquitination signals colocalize in cells in the form of cytoplasmic foci [69,105,106]. This supports the possibility that the ADPr-Ub hybrid modification of host and perhaps also of virus proteins or nucleic acids happens in cells. Interestingly, many viruses, including SARS-CoV-2, encode both ADPr and ubiquitination hydrolases [107]. Thus, PARP14/DTX3L-mediated ADPr-Ub may be an antiviral modification that is used to suppress the virus by an unknown mechanism, and the virus in this warfare possibly uses Mac1 (ADP-ribosyl hydrolase) and PLpro (deubiquitinase) enzymes to counteract the antiviral defense (Figure 3) [107,108]. PARP10 is another poorly characterized interferon-induced PARP. PARP10 was shown to be involved in suppression of alphavirus replication [109,110] and replication stress response [111–113] and could act similarly as PARP14 to work together with Deltex family E3s to produce ADPr-Ub in the fight against viruses. Another interferon-induced PARP – PARP7 co-operates with DTX2 to make the ADPr-Ub dual modification on the androgen receptor, which was proposed as a degron to control negative feedback of androgen [114].

## Additional complexity of ADPr-Ub

The study of ADPr-Ub is still in its infancy, with many mechanistic and functional aspects yet to be fully resolved. However, recent advances have begun to peel back the layers of this enigmatic modification, revealing a regulatory landscape far more intricate than initially anticipated. RNF114 exemplifies this regulatory complexity, serving as a reader and extender of ADPr-Ub [87]. Its tandem zinc fingers (ZnFs) and ubiquitin interacting motif (UIM) domain specifically recognize ADPr-Ub with a low micromolar affinity (Kd), indicative of a specific and potentially regulated interaction, while its RING domain further catalyzes K11-linked Ub chain elongation [87] (Figure 2C). The Ub hydrolase enzymes, such as Cezanne, potentially counteract this modification by selectively removing K11-linked Ub chains, providing a plausible mechanism for dynamic regulation of ADPr-Ub signaling in cells [87]. This dual functionality – reader and extender of ADPr-Ub – might extend to RNF114 paralogues (RNF125, RNF138, and RNF166), which share a similar domain architecture. Indeed, ADPr-Ub binding ability has been recently validated for RNF138 and RNF166 [115,116]. However, the molecular basis of ADPr-Ub recognition by the combined ZnF and UIM remains incompletely understood. While Ub can be reliably modeled into the UIM motif, the structural detail of ADP-ribose binding by the ZnF domain is less clear. Notably, their ZnF domains contain a conserved tyrosine residue that is predicted to stack with the adenine ring of ADP-ribose [78], which may be analogous to the critical tyrosine residue-mediated interaction observed in poly(ADP-ribose) recognition by PBZ ZnF modules in DDR proteins APLF (Aprataxin-PNK-like factor) and CHFR [59,60]. Our recent study suggests that additional readers of ADPr-Ub beyond RNF114, RNF166, and RNF138 may exist [87].

Notably, cellular studies demonstrate that both RNF114 and RNF166 extend ADPr-Ub modifications initially deposited by PARP7, PARP10, and tankyrase PARPs [29,115,116]. These extended ADPr-Ub K11 chains on protein substrates are proposed to mediate either their degradation [117] (e.g. CEPBP transcriptional regulator) or stabilization (e.g. tankyrases 1/2) [116], depending on cellular context. Thus, the activity of RNF114 and RNF166 that extends the ADPr-Ub may have important physiological functions. Notably, RNF114 was shown to be involved in DDR, telomere maintenance, and PARP trapping [118,119]. Furthermore, RNF114, RNF166, and RNF138 have been linked to antiviral immunity and inflammatory signaling [120–122]. Taken altogether, the emerging notion is that ADPr-Ub and its further ubiquitination may represent a versatile hybrid PTM deeply integrated into immune and genome surveillance networks, but further studies are needed to clarify this.

## Conclusion and perspectives

The recent discovery of the ADPr-Ub dual modification generated by PARPs and Deltex family E3s, sometimes in conjunction with RNF114 and its paralogues, suggests a novel mechanism for how the interplay between ADPr and ubiquitination could regulate cellular processes [23]. Although *in vitro* and cellular studies have confirmed the biochemical feasibility of ADPr ubiquitination, its precise cellular roles and regulatory mechanisms remain largely unexplored. Future research on ADPr ubiquitination should focus on several key directions. One major goal is determining its physiological relevance and identifying physiological substrates and downstream effectors of this dual modification. Currently, a critical barrier to advancing this field lies in the absence of robust cellular detection approaches; thus, developing specialized tools, such as engineered antibodies recognizing ADPr-Ub or chemical probes, will be essential for tracking ADPr ubiquitination in cells and tissues, ultimately advancing our understanding of its biological roles.

Recently, ADPr-Ub was detected for PARP10 in cells using a combination of antibodys-based Western blotting and supporting biochemical and cellular profiling [29]. Due to the liability of the ester linkage between ADPr and Ub [123], chemically synthesized non-hydrolysable ADPr-Ub analogs^78^ could greatly aid the discovery of ‘readers’ or ‘hydrolases’ of this dual ADPr-Ub modification and enable structural studies, directly capturing molecular interactions between ADPr-Ub and its ‘readers’ and ‘hydrolases’. These insights are critical for mapping binding interfaces and informing therapeutic strategies targeting ADPr-Ub signaling.

Advanced proteomics techniques, such as mass spectrometry-based methods capable of distinguishing this hybrid ADPr-Ub modification from conventional ubiquitination or ADP-ribosylation events [88], will be instrumental in uncovering endogenous targets of Deltex E3 ligases. Another important avenue of research is exploring the interplay between ADPr ubiquitination and other cellular signaling pathways. Given that both ubiquitination and ADP-ribosylation play central roles in genome stability, inflammation, and cellular metabolism, investigating how ADPr ubiquitination integrates into these pathways could reveal new regulatory networks with broad physiological implications.

## Abbreviations

ADPr: ADP-ribosylation
DDR: DNA damage response
E1: ubiquitin-activating enzyme
E2: ubiquitin-conjugating enzyme
E3: ubiquitin ligase
HECT: Homologous to the E6-AP Carboxyl Terminus
LUBAC: linear ubiquitin chain assembly complex
NAD^+^: nicotinamide adenine dinucleotide
PAR: poly-ADP-ribosylation
PBZ: PAR-binding zinc finger
PTM: post-translational modification
RBR: RING-between-RING
RING: Really Interesting New Gene
UIM: ubiquitin interacting motif
Ub: ubiquitin
ZnF: zinc fingers

## References

[1] Oh E. Akopian D. Rape M 2018 Principles of ubiquitin-dependent signaling Annu. Rev. Cell Dev. Biol. 34 137 162 10.1146/annurev-cellbio-100617-062802 30110556

[2] Pickart C.M. Eddins MJ 2004 Ubiquitin: structures, functions, mechanisms Biochim. Biophys. Acta 1695 55 72 10.1016/j.bbamcr.2004.09.019 15571809

[3] Ciechanover A. Heller H. Katz-Etzion R. Hershko A 1981 Activation of the heat-stable polypeptide of the ATP-dependent proteolytic system Proc. Natl. Acad. Sci. U.S.A. 78 761 765 10.1073/pnas.78.2.761 6262770 PMC319882

[4] Hershko A. Ciechanover A. Rose IA 1981 Identification of the active amino acid residue of the polypeptide of ATP-dependent protein breakdown J. Biol. Chem. 256 1525 1528 6257674

[5] Yau R. Rape M 2016 The increasing complexity of the ubiquitin code Nat. Cell Biol. 18 579 586 10.1038/ncb3358 27230526

[6] Hershko A. Ciechanover A 1998 The ubiquitin system Annu. Rev. Biochem. 67 425 479 10.1146/annurev.biochem.67.1.425 9759494

[7] Komander D. Rape M 2012 The ubiquitin code Annu. Rev. Biochem. 81 203 229 10.1146/annurev-biochem-060310-170328 22524316

[8] Thrower J.S. Hoffman L. Rechsteiner M. Pickart CM 2000 Recognition of the polyubiquitin proteolytic signal EMBO J. 19 94 102 10.1093/emboj/19.1.94 10619848 PMC1171781

[9] Pickart C.M 2001 Mechanisms underlying ubiquitination Annu. Rev. Biochem. 70 503 533 10.1146/annurev.biochem.70.1.503 11395416

[10] Madiraju C. Novack J.P. Reed J.C. Matsuzawa S.I 2022 K63 ubiquitination in immune signaling Trends Immunol 43 148 162 10.1016/j.it.2021.12.005 35033428 PMC8755460

[11] Hu H. Sun SC 2016 Ubiquitin signaling in immune responses Cell Res. 26 457 483 10.1038/cr.2016.40 27012466 PMC4822134

[12] Chen R.H. Chen Y.H. Huang TY 2019 Ubiquitin-mediated regulation of autophagy J. Biomed. Sci. 26 80 10.1186/s12929-019-0569-y 31630678 PMC6802350

[13] Erpapazoglou Z. Walker O. Haguenauer-Tsapis R 2014 Versatile roles of k63-linked ubiquitin chains in trafficking Cells 3 1027 1088 10.3390/cells3041027 25396681 PMC4276913

[14] Tracz M. Bialek W 2021 Beyond K48 and K63: non-canonical protein ubiquitination Cell. Mol. Biol. Lett. 26 1 10.1186/s11658-020-00245-6 33402098 PMC7786512

[15] Yang Q. Zhao J. Chen D. Wang Y 2021 E3 ubiquitin ligases: styles, structures and functions Mol. Biomed. 2 23 10.1186/s43556-021-00043-2 35006464 PMC8607428

[16] Morreale F.E. Walden H 2016 Types of Ubiquitin Ligases Cell 165 248 248 10.1016/j.cell.2016.03.003 27015313

[17] Buetow L. Huang DT 2016 Structural insights into the catalysis and regulation of E3 ubiquitin ligases Nat. Rev. Mol. Cell Biol. 17 626 642 10.1038/nrm.2016.91 27485899 PMC6211636

[18] Pao K.-C. Wood N.T. Knebel A. Rafie K. Stanley M. Mabbitt P.D. et al 2018 Activity-based E3 ligase profiling uncovers an E3 ligase with esterification activity Nature 556 381 385 10.1038/s41586-018-0026-1 29643511

[19] Simpson B.W. Trent MS 2019 Pushing the envelope: LPS modifications and their consequences Nat. Rev. Microbiol. 17 403 416 10.1038/s41579-019-0201-x 31142822 PMC6913091

[20] Ahel J. Lehner A. Vogel A. Schleiffer A. Meinhart A. Haselbach D. et al 2020 Moyamoya disease factor RNF213 is a giant E3 ligase with a dynein-like core and a distinct ubiquitin-transfer mechanism Elife 9 e56185 10.7554/eLife.56185 32573437 PMC7311170

[21] Rape M 2018 Ubiquitylation at the crossroads of development and disease Nat. Rev. Mol. Cell Biol. 19 59 70 10.1038/nrm.2017.83 28928488

[22] Otten E.G. Werner E. Crespillo-Casado A. Boyle K.B. Dharamdasani V. Pathe C. et al 2021 Ubiquitylation of lipopolysaccharide by RNF213 during bacterial infection Nature 594 111 116 10.1038/s41586-021-03566-4 34012115 PMC7610904

[23] Zhu K. Suskiewicz M.J. Hloušek-Kasun A. Meudal H. Mikoč A. Aucagne V. et al 2022 DELTEX E3 ligases ubiquitylate ADP-ribosyl modification on protein substrates Sci. Adv. 8 eadd4253 10.1126/sciadv.add4253 36197986 PMC7615817

[24] Kelsall I.R. McCrory E.H. Xu Y. Scudamore C.L. Nanda S.K. Mancebo-Gamella P. et al 2022 HOIL-1 ubiquitin ligase activity targets unbranched glucosaccharides and is required to prevent polyglucosan accumulation EMBO J. 41 e109700 10.15252/embj.2021109700 35274759 PMC9016349

[25] Sakamaki J.I. Ode K.L. Kurikawa Y. Ueda H.R. Yamamoto H. Mizushima N 2022 Ubiquitination of phosphatidylethanolamine in organellar membranes Mol. Cell 82 3677 3692 10.1016/j.molcel.2022.08.008 36044902

[26] Zhu K. Chatrin C. Suskiewicz M.J. Aucagne V. Foster B. Kessler B.M. et al 2024 Ubiquitylation of nucleic acids by DELTEX ubiquitin E3 ligase DTX3L EMBO Rep. 25 4172 4189 10.1038/s44319-024-00235-1 39242775 PMC11467253

[27] Dearlove E.L. Chatrin C. Buetow L. Ahmed S.F. Schmidt T. Bushell M. et al 2024 DTX3L ubiquitin ligase ubiquitinates single-stranded nucleic acids Elife 13 RP98070 10.7554/eLife.98070 39377462 PMC11460948

[28] Zhu K. Suskiewicz M.J. Chatrin C. Strømland Ø. Dorsey B.W. Aucagne V. et al 2024 DELTEX E3 ligases ubiquitylate ADP-ribosyl modification on nucleic acids Nucleic Acids Res. 52 801 815 10.1093/nar/gkad1119 38000390 PMC10810221

[29] Bejan D.S. Lacoursiere R.E. Pruneda J.N. Cohen MS 2025 Ubiquitin is directly linked via an ester to protein-conjugated mono-ADP-ribose EMBO J. 44 2211 2231 10.1038/s44318-025-00391-7 40000907 PMC12000418

[30] Barbour H. Nkwe N.S. Estavoyer B. Messmer C. Gushul-Leclaire M. Villot R. et al 2023 An inventory of crosstalk between ubiquitination and other post-translational modifications in orchestrating cellular processes iScience 26 106276 10.1016/j.isci.2023.106276 37168555 PMC10165199

[31] Matsumoto Y. Rottapel R 2023 PARsylation-mediated ubiquitylation: lessons from rare hereditary disease Cherubism Trends Mol. Med 29 390 405 10.1016/j.molmed.2023.02.001 36948987

[32] DaRosa P.A. Wang Z. Jiang X. Pruneda J.N. Cong F. Klevit R.E. et al 2015 Allosteric activation of the RNF146 ubiquitin ligase by a poly(ADP-ribosyl)ation signal Nature 517 223 226 10.1038/nature13826 25327252 PMC4289021

[33] Perina D. Mikoč A. Ahel J. Ćetković H. Žaja R. Ahel I 2014 Distribution of protein poly(ADP-ribosyl)ation systems across all domains of life DNA Repair (Amst.) 23 4 16 10.1016/j.dnarep.2014.05.003 24865146 PMC4245714

[34] Suskiewicz M.J. Prokhorova E. Rack J.G.M. Ahel I 2023 ADP-ribosylation from molecular mechanisms to therapeutic implications Cell 186 4475 4495 10.1016/j.cell.2023.08.030 37832523 PMC10789625

[35] Schuller M. Butler R.E. Ariza A. Tromans-Coia C. Jankevicius G. Claridge T.D.W. et al 2021 Molecular basis for DarT ADP-ribosylation of a DNA base Nature 596 597 602 10.1038/s41586-021-03825-4 34408320

[36] Groslambert J. Prokhorova E. Ahel I 2021 ADP-ribosylation of DNA and RNA DNA Repair (Amst.) 105 103144 10.1016/j.dnarep.2021.103144 34116477 PMC8385414

[37] Munnur D. Bartlett E. Mikolčević P. Kirby I.T. Rack J.G.M. Mikoč A. et al 2019 Reversible ADP-ribosylation of RNA Nucleic Acids Res. 47 5658 5669 10.1093/nar/gkz305 31216043 PMC6582358

[38] Zarkovic G. Belousova E.A. Talhaoui I. Saint-Pierre C. Kutuzov M.M. Matkarimov B.T. et al 2018 Characterization of DNA ADP-ribosyltransferase activities of PARP2 and PARP3: new insights into DNA ADP-ribosylation Nucleic Acids Res. 46 2417 2431 10.1093/nar/gkx1318 29361132 PMC5861426

[39] Weixler L. Feijs K.L.H. Zaja R 2022 ADP-ribosylation of RNA in mammalian cells is mediated by TRPT1 and multiple PARPs Nucleic Acids Res. 50 9426 9441 10.1093/nar/gkac711 36018800 PMC9458441

[40] Talhaoui I. Lebedeva N.A. Zarkovic G. Saint-Pierre C. Kutuzov M.M. Sukhanova M.V. et al 2016 Poly(ADP-ribose) polymerases covalently modify strand break termini in DNA fragments in vitro Nucleic Acids Res. 44 9279 9295 10.1093/nar/gkw675 27471034 PMC5100588

[41] Cohen M.S. Chang P 2018 Insights into the biogenesis, function, and regulation of ADP-ribosylation Nat. Chem. Biol. 14 236 243 10.1038/nchembio.2568 29443986 PMC5922452

[42] Gupte R. Liu Z. Kraus WL 2017 PARPs and ADP-ribosylation: recent advances linking molecular functions to biological outcomes Genes Dev. 31 101 126 10.1101/gad.291518.116 28202539 PMC5322727

[43] Lüscher B. Ahel I. Altmeyer M. Ashworth A. Bai P. Chang P. et al 2022 ADP-ribosyltransferases, an update on function and nomenclature FEBS J. 289 7399 7410 10.1111/febs.16142 34323016 PMC9027952

[44] Suskiewicz M.J. Munnur D. Strømland Ø. Yang J.-C. Easton L.E. Chatrin C. et al 2023 Updated protein domain annotation of the PARP protein family sheds new light on biological function Nucleic Acids Res. 51 8217 8236 10.1093/nar/gkad514 37326024 PMC10450202

[45] Vyas S. Matic I. Uchima L. Rood J. Zaja R. Hay R.T. et al 2014 Family-wide analysis of poly(ADP-ribose) polymerase activity Nat. Commun. 5 4426 10.1038/ncomms5426 25043379 PMC4123609

[46] Chen Q. Kassab M.A. Dantzer F. Yu X 2018 PARP2 mediates branched poly ADP-ribosylation in response to DNA damage Nat. Commun. 9 3233 10.1038/s41467-018-05588-5 30104678 PMC6089979

[47] Miwa M. Saikawa N. Yamaizumi Z. Nishimura S. Sugimura T 1979 Structure of poly(adenosine diphosphate ribose): identification of 2’-[1’’-ribosyl-2’’-(or 3’’-)(1’’’-ribosyl)]adenosine-5’,5’’,5’’’-tris(phosphate) as a branch linkage Proc. Natl. Acad. Sci. U.S.A 76 595 599 10.1073/pnas.76.2.595 218210 PMC382995

[48] Rack J.G.M. Palazzo L. Ahel I 2020 (ADP-ribosyl)hydrolases: structure, function, and biology Genes Dev. 34 263 284 10.1101/gad.334631.119 32029451 PMC7050489

[49] Slade D. Dunstan M.S. Barkauskaite E. Weston R. Lafite P. Dixon N. et al 2011 The structure and catalytic mechanism of a poly(ADP-ribose) glycohydrolase Nature 477 616 620 10.1038/nature10404 21892188 PMC3184140

[50] Rack J.G.M. Liu Q. Zorzini V. Voorneveld J. Ariza A. Honarmand Ebrahimi K. et al 2021 Mechanistic insights into the three steps of poly(ADP-ribosylation) reversal Nat. Commun. 12 4581 10.1038/s41467-021-24723-3 34321462 PMC8319183

[51] Brochu G. Duchaine C. Thibeault L. Lagueux J. Shah G.M. Poirier GG 1994 Mode of action of poly(ADP-ribose) glycohydrolase Biochim. Biophys. Acta 1219 342 350 10.1016/0167-4781(94)90058-2 7918631

[52] Lin W. Amé J.-C. Aboul-Ela N. Jacobson E.L. Jacobson M.K 1997 Isolation and Characterization of the cDNA Encoding Bovine Poly(ADP-ribose) Glycohydrolase Journal of Biological Chemistry 272 11895 11901 10.1074/jbc.272.18.11895 9115250

[53] Fontana P. Bonfiglio J.J. Palazzo L. Bartlett E. Matic I. Ahel I 2017 Serine ADP-ribosylation reversal by the hydrolase ARH3 Elife 6 10.7554/eLife.28533 PMC5552275 28650317

[54] Sharifi R. Morra R. Appel C.D. Tallis M. Chioza B. Jankevicius G. et al 2013 Deficiency of terminal ADP-ribose protein glycohydrolase TARG1/C6orf130 in neurodegenerative disease EMBO J. 32 1225 1237 10.1038/emboj.2013.51 23481255 PMC3642678

[55] Tromans-Coia C. Sanchi A. Moeller G.K. Timinszky G. Lopes M. Ahel I 2021 TARG1 protects against toxic DNA ADP-ribosylation Nucleic Acids Res. 49 10477 10492 10.1093/nar/gkab771 34508355 PMC8501950

[56] Wondisford A.R. Lee J. Lu R. Schuller M. Groslambert J. Bhargava R. et al 2024 Deregulated DNA ADP-ribosylation impairs telomere replication Nat. Struct. Mol. Biol. 31 791 800 10.1038/s41594-024-01279-6 38714889 PMC11102865

[57] Rosenthal F. Feijs K.L.H. Frugier E. Bonalli M. Forst A.H. Imhof R. et al 2013 Macrodomain-containing proteins are new mono-ADP-ribosylhydrolases Nat. Struct. Mol. Biol. 20 502 507 10.1038/nsmb.2521 23474714

[58] Teloni F. Altmeyer M 2016 Readers of poly(ADP-ribose): designed to be fit for purpose Nucleic Acids Res. 44 993 1006 10.1093/nar/gkv1383 26673700 PMC4756826

[59] Ahel I. Ahel D. Matsusaka T. Clark A.J. Pines J. Boulton S.J. et al 2008 Poly(ADP-ribose)-binding zinc finger motifs in DNA repair/checkpoint proteins Nature 451 81 85 10.1038/nature06420 18172500

[60] Eustermann S. Brockmann C. Mehrotra P.V. Yang J.-C. Loakes D. West S.C. et al 2010 Solution structures of the two PBZ domains from human APLF and their interaction with poly(ADP-ribose) Nat. Struct. Mol. Biol. 17 241 243 10.1038/nsmb.1747 20098424 PMC2912505

[61] Karras G.I. Kustatscher G. Buhecha H.R. Allen M.D. Pugieux C. Sait F. et al 2005 The macro domain is an ADP-ribose binding module EMBO J. 24 1911 1920 10.1038/sj.emboj.7600664 15902274 PMC1142602

[62] Rack J.G.M. Perina D. Ahel I 2016 Macrodomains: Structure, Function, Evolution, and Catalytic Activities Annu. Rev. Biochem. 85 431 454 10.1146/annurev-biochem-060815-014935 26844395

[63] Forst A.H. Karlberg T. Herzog N. Thorsell A.-G. Gross A. Feijs K.L.H et al 2013 Recognition of mono-ADP-ribosylated ARTD10 substrates by ARTD8 macrodomains Structure 21 462 475 10.1016/j.str.2012.12.019 23473667

[64] Aravind L 2001 The WWE domain: a common interaction module in protein ubiquitination and ADP ribosylation Trends Biochem. Sci. 26 273 275 10.1016/s0968-0004(01)01787-x 11343911

[65] Gatti M. Imhof R. Huang Q. Baudis M. Altmeyer M 2020 The Ubiquitin Ligase TRIP12 Limits PARP1 Trapping and Constrains PARP Inhibitor Efficiency Cell Rep. 32 107985 10.1016/j.celrep.2020.107985 32755579 PMC7408484

[66] Kashima L. Idogawa M. Mita H. Shitashige M. Yamada T. Ogi K. et al 2012 CHFR protein regulates mitotic checkpoint by targeting PARP-1 protein for ubiquitination and degradation J. Biol. Chem. 287 12975 12984 10.1074/jbc.M111.321828 22337872 PMC3339944

[67] Wang L. Sun X. He J. Liu Z 2021 Functions and molecular mechanisms of deltex Family Ubiquitin E3 Ligases in Development and Disease Front. Cell Dev. Biol. 9 706997 10.3389/fcell.2021.706997 34513839 PMC8424196

[68] Wang Z. Michaud G.A. Cheng Z. Zhang Y. Hinds T.R. Fan E. et al 2012 Recognition of the *iso* -ADP-ribose moiety in poly(ADP-ribose) by WWE domains suggests a general mechanism for poly(ADP-ribosyl)ation-dependent ubiquitination . Genes Dev. 26 235 240 10.1101/gad.182618.111 22267412 PMC3278890

[69] Kar P. Chatrin C. Đukić N. Suyari O. Schuller M. Zhu K. et al 2024 PARP14 and PARP9/DTX3L regulate interferon-induced ADP-ribosylation EMBO J. 43 2929 2953 10.1038/s44318-024-00126-0 38834853 PMC11251020

[70] Oberoi J. Richards M.W. Crumpler S. Brown N. Blagg J. Bayliss R 2010 Structural Basis of Poly(ADP-ribose) Recognition by the Multizinc Binding Domain of Checkpoint with Forkhead-associated and RING Domains (CHFR) Journal of Biological Chemistry 285 39348 39358 10.1074/jbc.M110.159855 20880844 PMC2998101

[71] Verheugd P. Forst A.H. Milke L. Herzog N. Feijs K.L.H. Kremmer E. et al 2013 Regulation of NF-κB signalling by the mono-ADP-ribosyltransferase ARTD10 Nat. Commun. 4 1683 10.1038/ncomms2672 23575687

[72] Zhang Y. Liu S. Mickanin C. Feng Y. Charlat O. Michaud G.A. et al 2011 RNF146 is a poly(ADP-ribose)-directed E3 ligase that regulates axin degradation and Wnt signalling Nat. Cell Biol. 13 623 629 10.1038/ncb2222 21478859

[73] Callow M.G. Tran H. Phu L. Lau T. Lee J. Sandoval W.N. et al 2011 Ubiquitin ligase RNF146 regulates tankyrase and Axin to promote Wnt signaling PLoS ONE 6 e22595 10.1371/journal.pone.0022595 21799911 PMC3143158

[74] Chang W. Dynek J.N. Smith S 2003 TRF1 is degraded by ubiquitin-mediated proteolysis after release from telomeres Genes Dev. 17 1328 1333 10.1101/gad.1077103 12782650 PMC196064

[75] Bhogaraju S. Kalayil S. Liu Y. Bonn F. Colby T. Matic I et al 2016 Phosphoribosylation of ubiquitin promotes serine ubiquitination and impairs conventional ubiquitination Cell 167 1636 1649 10.1016/j.cell.2016.11.019 27912065

[76] Qiu J. Sheedlo M.J. Yu K. Tan Y. Nakayasu E.S. Das C. et al 2016 Ubiquitination independent of E1 and E2 enzymes by bacterial effectors Nature 533 120 124 10.1038/nature17657 27049943 PMC4905768

[77] Wang T. Song X. Tan J. Xian W. Zhou X. Yu M. et al 2024 Legionella effector LnaB is a phosphoryl-AMPylase that impairs phosphosignalling Nature 631 393 401 10.1038/s41586-024-07573-z 38776962

[78] Fu J. Li S. Guan H. Li C. Zhao Y.-B. Chen T.-T. et al 2024 Legionella maintains host cell ubiquitin homeostasis by effectors with unique catalytic mechanisms Nat. Commun. 15 10.1038/s41467-024-50311-2 PMC11251166 39009586

[79] Shin D. Mukherjee R. Liu Y. Gonzalez A. Bonn F. Liu Y et al 2020 Regulation of phosphoribosyl-linked serine ubiquitination by deubiquitinases DupA and DupB Mol. Cell 77 164 179 10.1016/j.molcel.2019.10.019 31732457 PMC6941232

[80] Yan F. Huang C. Wang X. Tan J. Cheng S. Wan M et al 2020 Threonine ADP-Ribosylation of Ubiquitin by a Bacterial Effector Family Blocks Host Ubiquitination Mol. Cell 78 641 652 10.1016/j.molcel.2020.03.016 32330457

[81] Takeyama K. Aguiar R.C.T. Gu L. He C. Freeman G.J. Kutok J.L. et al 2003 The BAL-binding protein BBAP and related Deltex family members exhibit ubiquitin-protein isopeptide ligase activity J. Biol. Chem. 278 21930 21937 10.1074/jbc.M301157200 12670957

[82] Yang C.-S. Jividen K. Spencer A. Dworak N. Ni L. Oostdyk L.T. et al 2017 Ubiquitin Modification by the E3 Ligase/ADP-Ribosyltransferase Dtx3L/Parp9 Mol. Cell 66 503 516 10.1016/j.molcel.2017.04.028 28525742 PMC5556935

[83] Suskiewicz M.J. Zobel F. Ogden T.E.H. Fontana P. Ariza A. Yang J.-C. et al 2020 HPF1 completes the PARP active site for DNA damage-induced ADP-ribosylation Nature 579 598 602 10.1038/s41586-020-2013-6 32028527 PMC7104379

[84] Chatrin C. Gabrielsen M. Buetow L. Nakasone M.A. Ahmed S.F. Sumpton D. et al 2020 Structural insights into ADP-ribosylation of ubiquitin by Deltex family E3 ubiquitin ligases Sci. Adv. 6 10.1126/sciadv.abc0418 PMC7500938 32948590

[85] Grunewald M.E. Chen Y. Kuny C. Maejima T. Lease R. Ferraris D. et al 2019 The coronavirus macrodomain is required to prevent PARP-mediated inhibition of virus replication and enhancement of IFN expression PLoS Pathog. 15 e1007756 10.1371/journal.ppat.1007756 31095648 PMC6521996

[86] Đukić N. Strømland Ø. Elsborg J.D. Munnur D. Zhu K. Schuller M. et al 2023 PARP14 is a PARP with both ADP-ribosyl transferase and hydrolase activities Sci. Adv. 9 eadi2687 10.1126/sciadv.adi2687 37703374 PMC10499325

[87] Kloet M.S. Chatrin C. Mukhopadhyay R. Van Tol B.D.M. Smith R. Rotman S.A et al 2025 Identification of RNF114 as ADPr-Ub reader through non-hydrolysable ubiquitinated ADP-ribose Nat. Commun 16 6319 10.1038/s41467-025-61111-7 40634336 PMC12241653

[88] Kolvenbach A. Palumbieri M.D. Colby T. Nadarajan D. Bode R. Matić I 2025 Serine ADPr on histones and PARP1 is a cellular target of ester-linked ubiquitylation Nat. Chem. Biol. 10.1038/s41589-025-01974-5 40634527 PMC12568645

[89] Ahmed S.F. Buetow L. Gabrielsen M. Lilla S. Chatrin C. Sibbet G.J. et al 2020 DELTEX2 C-terminal domain recognizes and recruits ADP-ribosylated proteins for ubiquitination Sci. Adv. 6 eabc0629 10.1126/sciadv.abc0629 32937373 PMC7442474

[90] Hedstrom L 2002 Serine protease mechanism and specificity Chem. Rev. 102 4501 4524 10.1021/cr000033x 12475199

[91] Rack J.G.M. Zorzini V. Zhu Z. Schuller M. Ahel D. Ahel I 2020 Viral macrodomains: a structural and evolutionary assessment of the pharmacological potential Open Biol. 10 200237 10.1098/rsob.200237 33202171 PMC7729036

[92] Kleine H. Poreba E. Lesniewicz K. Hassa P.O. Hottiger M.O. Litchfield D.W. et al 2008 Substrate-assisted catalysis by PARP10 limits its activity to mono-ADP-ribosylation Mol. Cell 32 57 69 10.1016/j.molcel.2008.08.009 18851833

[93] Kelly M. Dietz C. Kasson S. Zhang Y. Holtzman M.J. Kim I.K 2024 Deltex family E3 ligases specifically ubiquitinate the terminal ADP-ribose of poly(ADP-ribosyl)ation Biochem. Biophys. Res. Commun 720 150101 10.1016/j.bbrc.2024.150101 38749191 PMC11219154

[94] Munnur D. Ahel I 2017 Reversible mono-ADP-ribosylation of DNA breaks FEBS J. 284 4002 4016 10.1111/febs.14297 29054115 PMC5725667

[95] Maris C. Dominguez C. Allain F.H.-T 2005 The RNA recognition motif, a plastic RNA-binding platform to regulate post-transcriptional gene expression FEBS J. 272 2118 2131 10.1111/j.1742-4658.2005.04653.x 15853797

[96] Saleh H. Liloglou T. Rigden D.J. Parsons J.L. Grundy GJ 2024 KH-like Domains in PARP9/DTX3L and PARP14 Coordinate Protein-Protein Interactions to Promote Cancer Cell Survival J. Mol. Biol. 436 168434 10.1016/j.jmb.2023.168434 38182103 PMC11080071

[97] Ashok Y. Vela-Rodríguez C. Yang C. Alanen H.I. Liu F. Paschal B.M. et al 2022 Reconstitution of the DTX3L-PARP9 complex reveals determinants for high-affinity heterodimerization and multimeric assembly Biochem. J. 479 289 304 10.1042/BCJ20210722 35037691

[98] Matsuno K. Diederich R.J. Go M.J. Blaumueller C.M. Artavanis-Tsakonas S 1995 Deltex acts as a positive regulator of Notch signaling through interactions with the Notch ankyrin repeats Development 121 2633 2644 10.1242/dev.121.8.2633 7671825

[99] Matsuno K. Eastman D. Mitsiades T. Quinn A.M. Carcanciu M.L. Ordentlich P. et al 1998 Human deltex is a conserved regulator of Notch signalling Nat. Genet. 19 74 78 10.1038/ng0598-74 9590294

[100] Matsuno K. Ito M. Hori K. Miyashita F. Suzuki S. Kishi N. et al 2002 Involvement of a proline-rich motif and RING-H2 finger of Deltex in the regulation of Notch signaling Development 129 1049 1059 10.1242/dev.129.4.1049 11861487

[101] Djerir B. Marois I. Dubois J.-C. Findlay S. Morin T. Senoussi I. et al 2024 An E3 ubiquitin ligase localization screen uncovers DTX2 as a novel ADP-ribosylation-dependent regulator of DNA double-strand break repair J. Biol. Chem. 300 107545 10.1016/j.jbc.2024.107545 38992439 PMC11345397

[102] Yan Q. Dutt S. Xu R. Graves K. Juszczynski P. Manis J.P. et al 2009 BBAP monoubiquitylates histone H4 at lysine 91 and selectively modulates the DNA damage response Mol. Cell 36 110 120 10.1016/j.molcel.2009.08.019 19818714 PMC2913878

[103] Zhang Y. Mao D. Roswit W.T. Jin X. Patel A.C. Patel D.A. et al 2015 PARP9-DTX3L ubiquitin ligase targets host histone H2BJ and viral 3C protease to enhance interferon signaling and control viral infection Nat. Immunol. 16 1215 1227 10.1038/ni.3279 26479788 PMC4653074

[104] Daugherty M.D. Young J.M. Kerns J.A. Malik HS 2014 Rapid evolution of PARP genes suggests a broad role for ADP-ribosylation in host-virus conflicts PLoS Genet. 10 e1004403 10.1371/journal.pgen.1004403 24875882 PMC4038475

[105] Ribeiro V.C. Russo L.C. Hoch NC 2024 PARP14 is regulated by the PARP9/DTX3L complex and promotes interferon γ-induced ADP-ribosylation EMBO J. 43 2908 2928 10.1038/s44318-024-00125-1 38834852 PMC11251048

[106] Raja R. Biswas B. Abraham R. Wang Y. Chang C.-Y. Hendriks I.A. et al 2025 Interferon-induced PARP14-mediated ADP-ribosylation in p62 bodies requires the ubiquitin-proteasome system EMBO J. 44 2741 2773 10.1038/s44318-025-00421-4 40195501 PMC12084362

[107] Fehr A.R. Channappanavar R. Jankevicius G. Fett C. Zhao J. Athmer J. et al 2016 The Conserved Coronavirus Macrodomain Promotes Virulence and Suppresses the Innate Immune Response during Severe Acute Respiratory Syndrome Coronavirus Infection MBio 7 e01721-16 10.1128/mBio.01721-16 27965448 PMC5156301

[108] Shin D. Mukherjee R. Grewe D. Bojkova D. Baek K. Bhattacharya A. et al 2020 Papain-like protease regulates SARS-CoV-2 viral spread and innate immunity Nature 587 657 662 10.1038/s41586-020-2601-5 32726803 PMC7116779

[109] Krieg S. Pott F. Potthoff L. Verheirstraeten M. Bütepage M. Golzmann A. et al 2023 Mono-ADP-ribosylation by PARP10 inhibits Chikungunya virus nsP2 proteolytic activity and viral replication Cell. Mol. Life Sci. 80 72 10.1007/s00018-023-04717-8 36840772 PMC9959937

[110] Atasheva S. Frolova E.I. Frolov I 2014 Interferon-stimulated poly(ADP-Ribose) polymerases are potent inhibitors of cellular translation and virus replication J. Virol. 88 2116 2130 10.1128/JVI.03443-13 24335297 PMC3911523

[111] Schleicher E.M. Galvan A.M. Imamura-Kawasawa Y. Moldovan G.L. Nicolae CM 2018 PARP10 promotes cellular proliferation and tumorigenesis by alleviating replication stress Nucleic Acids Res. 46 8908 8916 10.1093/nar/gky658 30032250 PMC6158756

[112] Khatib J.B. Dhoonmoon A. Moldovan G.-L. Nicolae CM 2024 PARP10 promotes the repair of nascent strand DNA gaps through RAD18 mediated translesion synthesis Nat. Commun. 15 6197 10.1038/s41467-024-50429-3 39043663 PMC11266678

[113] Shahrour M.A. Nicolae C.M. Edvardson S. Ashhab M. Galvan A.M. Constantin D et al 2016 PARP10 deficiency manifests by infantile neurodegeneration and DNA repair defect Neurogenetics 17 227 10.1007/s10048-016-0493-1 27624574 PMC5096377

[114] Wierbiłowicz K. Yang C.-S. Almaghasilah A. Wesołowski P.A. Pracht P. Dworak N.M. et al 2025 Parp7 generates an ADP-ribosyl degron that controls negative feedback of androgen signaling EMBO J. 44 4720 4744 10.1038/s44318-025-00510-4 40681873 PMC12402299

[115] Lacoursiere R.E. Upadhyaya K. Sidhu J.K. Bejan D.S. Siordia I.R. Cohen M.S. et al 2025 A family of E3 ligases extend K11 polyubiquitin on sites of MARUbylation bioRxiv 2025 11 2025.05.11.653360 10.1101/2025.05.11.653360 40463042 PMC12132378

[116] Perrard J. Gao K. Ring K. Smith S Deltex and RING-UIM E3 ligases cooperate to create a ubiquitin-ADP-ribose hybrid mark on tankyrase, promoting its stabilization Biochemistry 2025 09 Biochemistry 10.1101/2025.04.09.648013 PMC12407064 40901936

[117] Stokes M.S. Kim Y.J. Kim Y. Koul S. Chiu S.-P. Dasovich M. et al 2025 NAD ^+^ Sensing by PARP7 Regulates the C/EBPβ-Dependent Transcription Program in Adipose Tissue In Vivo bioRxiv 2025.04.07.647692 10.1101/2025.04.07.647692 40291749 PMC12027069

[118] Longarini E.J. Dauben H. Locatelli C. Wondisford A.R. Smith R. Muench C et al 2023 Modular antibodies reveal DNA damage-induced mono-ADP-ribosylation as a second wave of PARP1 signaling Mol. Cell 83 1743 1760 10.1016/j.molcel.2023.03.027 37116497 PMC10205078

[119] Li P. Zhen Y. Kim C. Liu Z. Hao J. Deng H. et al 2023 Nimbolide targets RNF114 to induce the trapping of PARP1 and synthetic lethality in *BRCA*-mutated cancer Sci. Adv. 9 eadg7752 10.1126/sciadv.adg7752 37878693 PMC10599614

[120] Zhang Y. Zhang H. Zheng G.-L. Yang Q. Yu S. Wang J. et al 2019 Porcine RING Finger Protein 114 Inhibits Classical Swine Fever Virus Replication via K27-Linked Polyubiquitination of Viral NS4B J. Virol. 93 01248 19 10.1128/JVI.01248-19 PMC6803260 31413123

[121] Chen H.W. Yang Y.K. Xu H. Yang W.W. Zhai Z.H. Chen DY 2015 Ring finger protein 166 potentiates RNA virus-induced interferon-β production via enhancing the ubiquitination of TRAF3 and TRAF6 Sci. Rep. 5 10.1038/srep14770 PMC4600972 26456228

[122] Arimoto K. Takahashi H. Hishiki T. Konishi H. Fujita T. Shimotohno K 2007 Negative regulation of the RIG-I signaling by the ubiquitin ligase RNF125 Proc. Natl. Acad. Sci. U.S.A 104 7500 7505 10.1073/pnas.0611551104 17460044 PMC1863485

[123] Longarini E.J. Matić I 2024 Preserving ester-linked modifications reveals glutamate and aspartate mono-ADP-ribosylation by PARP1 and its reversal by PARG Nat. Commun. 15 4239 10.1038/s41467-024-48314-0 38762517 PMC11102441

